# See Spot save lives: fear, humanitarianism, and war in the development of robot quadrupeds

**DOI:** 10.1057/s42984-021-00037-y

**Published:** 2021-11-24

**Authors:** Jeremy Moses, Geoffrey Ford

**Affiliations:** Department of Political Science and International Relations, University of Canterbury, Private Bag 4800, Christchurch, New Zealand

**Keywords:** Military robotics, Robot quadrupeds, Humanitarianism, Sentiment analysis, Lethal autonomous weapons, Killer robots

## Abstract

Boston Dynamics’ robotic quadrupeds have achieved infamy and virality through a series of social media videos since 2008. In 2019 Boston Dynamics began commercial sale of ‘Spot’, a moving, sensing, networked robot dog. Spot has been designed to be a platform, which can be augmented with hardware payloads (e.g. sensors, robotic arm) and software to command Spot to conduct specific missions. In this paper we first trace the development of Spot and highlight the interest of the United States military in its development. This is followed by our text analysis of social media reactions to Boston Dynamics’ quadrupeds, revealing public fascination as well as ongoing suspicion and dark humour about ‘killer robots’. We then discuss how humanitarian applications, including in response to the COVID-19 pandemic, have been used as an opportunity to promote Spot and overcome public negativity. This is an example of a more general strategy advocates use to garner acceptance for autonomous robots in both civilian and military roles using humanitarian justifications: the robots ‘save lives.’ We conclude by discussing how Spot and other robot quadrupeds demonstrate the intertwining of humanitarian and military applications in the development, normalization and deployment of autonomous robots.

## Introduction

Where is Spot? And by Spot, we don’t mean the cute yellow puppy from the famous childrens’ books by Eric Hill, but the yellow robotic one designed by Boston Dynamics and released for public sale in 2019. In recent months, Boston Dynamics’ Spot seems to have popped up in a variety of places. Over recent years publicity videos have revealed Spot pulling a rickshaw (Adam Savage’s Tested 2020), herding sheep (Rocos—Robot Operations Platform 2020), climbing stairs (Aker BP ASA 2020), and dancing (Boston Dynamics 2018c). Spot’s also been seen conducting a surveillance missions on factory floors and building sites (Boston Dynamics 2018b) and working alongside the NYPD (Stephen 2021). In these videos, Spot comes across as a cute, slick piece of robotic technology that can perform some impressive moves and mechanical tasks in commercial and domestic environments. But it hasn’t always been thus. Earlier publicity videos released by Boston Dynamics presented a somewhat more menacing picture, particularly the 2018 video of a pair of Spots, with their whirring, rhythmic gait, working together to open and enter a door (Boston Dynamics 2018a). It is these images that have engaged, enthralled, and sometimes terrified a global audience, with the aforementioned YouTube video having been viewed over 140 million times and Boston Dynamics currently holding just under three million subscribers on that platform alone.

While Boston Dynamics has been at the forefront of the development of legged robots since the early 1990s and is the most recognizable company working in this area, they are now operating in a more crowded and competitive field. Amongst the other tech firms working on variations of robot quadrupeds are Ghost Robotics (on which more later), the MIT Biomimetics Lab, the Swiss firm Anybotics, and Chinese companies Deep Robotics and Weilan. Interestingly, despite Boston Dynamics appearing to have spent much of the last five years gearing Spot more toward a commercial or industrial market, it is Spot that has become something of a public touchstone for debates over the development and deployment of artificially intelligent robots for military and security purposes. At present, however, most of Spot’s applications are remote controlled (very much maintaining a ‘human in the loop’) and it has not yet been demonstrated or used as a platform for the exercise of lethal force.

So how does Spot fit into the debate over lethal autonomous weapons systems (LAWS)? As with many current robotic systems, Spot has been designed as a multi-application platform, leaving open the possibility of greater autonomy and lethality, depending on how the end user adapts its software and hardware. It is precisely this characteristic of Spot (and other emergent robotic quadrupeds) as *platforms* for multiple potential end-user applications that is of interest here, as this leaves open the possibility of the technology being imagined and used in ways that range from the benignly humanitarian to the aggressively militaristic and everything in between. The recent promotion of a gun-bearing robotic quadruped by Ghost Robotics, which will be discussed later in the article, has shed further light on this issue and illustrates how robotics companies are both courting and distancing themselves from military contracts at the same time as they seek to manage the public image of their companies and their products.

Spot is the focus of this paper due to the enormous public interest it has generated and the connections between it and the *potential* for lethal autonomy that has conjured fear in the public mind. Understanding how and for what purposes the technology was developed, how the public has reacted to its emergence, and how its producers have responded to negative public sentiment tells us a lot about the ways in which discourses of technology and future war are managed. It is also a relevant case study for understanding how the image of future LAWS developments might be managed as more militaries seek technological advantages over their rivals.

To get at some of these issues in this article, we first offer a brief history of the development of Spot, with emphasis on its genesis as a Defense Advanced Research Projects Agency (DARPA)-funded project. We then present the method and analysis used to examine the intense and varied public sentiment about Spot on Twitter, showing that there is a mix of awe and terror in public responses. This is followed by an overview of Boston Dynamics’ attempt to refocus attention on the purported humanitarian benefits of their technology. Here, the discussion focuses on the turn toward representation of Spot as technology that can ‘save lives’ through management and surveillance of dangerous workplaces, and operations in dangerous environments that are inhospitable to human activity, including collapsed buildings, radioactive environments, chemical spills, and pandemic response.

The final section will then demonstrate that the promotion of a humane/humanitarian image for Spot is closely related to the military applications that have been envisaged for such robots since at least the 1990s. We argue that the claim that robot quadrupeds can ‘save lives’ in hostile environments runs across the military and humanitarian discourse, obscuring and potentially alleviating the sense of threat that these technologies represent in the public imagination. The recent moves toward painting these technologies with a kinder, more humanitarian face is not, therefore, an indication that they are not being developed with future war-fighting in mind. The aforementioned emergence of Ghost Robotics as a major player in the military field represents a significant example of this and will be discussed in the latter parts of this article.

The arguments on the intersections of humanitarianism and war in this article are related to the extensive literature on this subject in international relations theory and history (Der Derian and James 2001; Jabri, 2007; Dillon and Reid, 2009; Moses, 2010; Moses, 2020). Recent works by Carvin and Williams (2014), Maja Zehfuss (2011, 2012, 2018), and Samuel Moyn (2021) are exemplars of this field, offering detailed theoretical and historical accounts of ways in which the discourses and practices of ‘humane’ war have sought to make war more palatable to liberal and humanitarian publics. More specifically, the article adds to the growing body of literature on the marketing of new ‘humanitarian’ technologies that span the civilian-military divide (for example, see: Sandvik and Lohne 2014; Kaplan forthcoming), reinforcing the concern that humanitarian discourse is being manipulated to downplay risks and emphasise potential benefits in the attempt win public support and attract further funding for these technologies. Drawing upon a combination of these influences and digital methods, we argue that these are trends that deserve greater awareness and wariness in the ongoing debates over lethal autonomous weapons.

### A brief history of Spot

A brief overview of the emergence of Spot is useful here, as it shows the path from military funding to commercialisation and the shifts in framing that have accompanied that process of development. Spot emerged through a series of iterations out of a US Defense Advanced Research Projects Agency (DARPA)-funded project to build a robot capable of hauling gear for soliders over terrains that would be challenging for wheeled or tracked vehicles. That initial project, named ‘BigDog’, was reportedly started in 2003 (Markoff 2013) and the first video of testing was released by Boston Dynamics in March, 2008 (Olinerd 2008). DARPA remained invested in the development of the Boston Dynamics quadruped for a number of years, and in 2010 sought the development of a bigger version of the 2008 BigDog (Brown 2010). This resulted in the development of the Legged Squad Support System (LS3), which was showcased in a DARPA video in 2012 (DARPAtv 2012), but was ultimately determined to be too noisy for military applications and was abandoned by DARPA by late 2015 (Hern 2015).

Over the period between 2009 and 2015, Boston Dynamics had continued to experiment with smaller and faster quadrupeds (named LittleDog, Cheetah, and Wildcat), again with significant support from DARPA (Boston Dynamics 2012; Boston Dynamics 2013; Boston Dynamics 2009). This research led to the first iteration of the ‘Spot’ line of quadrupeds in February, 2015; a medium-sized, battery-powered version that was quieter, more agile, and faster than the BigDog line. This version of Spot was tested in training with the US Marine Corps Rifle Squad in late 2015, being used for reconnaissance and surveillance ahead of a room-clearing exercise (SciNews 2015). This appears to be the last time that Boston Dynamics robots were used by the US military, though in 2021 Spot was used in a military training exercise in France and was controversially trialed by US police forces in Massachusetts in 2019 and New York in 2021 (Vincent 2021; Jarmanning 2019; Stephen 2021). Perceptions of dystopian danger posed by the development of robot quadrupeds have persisted, both in response to these military and police trials of Spot and due to deeper negative sentiment toward robots that is often reflected in science fiction and popular culture. The release of the ‘Metalhead’ episode of *Black Mirror* in late 2017, for example, depicting a bleak futuristic landscape populated by ruthless lethal autonomous quadrupeds, was inspired by Boston Dynamics’ videos (Hibberd 2017).

It appears, however, that Boston Dynamics are keen to distance themselves from such dystopian images of the future being related to their technology. Over the period since 2010 a series of changes in corporate ownership appeared to point away from the military market and towards a greater consumer and commercial focus. In 2013, Boston Dynamics was bought by Google parent company Alphabet Inc, which was seeking to expand its research and development of consumer robotics. At the time of the sale, it was made clear that under Google, Boston Dynamics ‘would honor existing military contracts, but that it did not plan to move toward becoming a military contractor on its own’(Markoff 2013). Only a few years later, an apparent diminution of interest in consumer robotics at Alphabet Inc led to the sale of Boston Dynamics to the Japanese tech investment firm Softbank in mid-2017 (Hern 2017). A further change in was announced in June 2021, with Hyundai taking majority control over the company. With the successive changes in ownership the re-positioning of Spot away from its military roots toward a more domestic and industrial focus was made quite explicitly. The partnership with Hyundai has been characterised as aiming ‘to develop advanced technologies that enhance people's lives and promote safety, thereby realizing progress for humanity’ (Boston Dynamics 2021).This move was reinforced by the clause in the leasing contracts for Spot (which by 2018 had taken on its now-familiar yellow form) stipulating that it cannot be used ‘to physically harm or intimidate people’ (Jarmanning 2019).

The variations in Boston Dynamics’ messaging prompted a range of questions: How has Boston Dynamics’ messaging around Spot changed over time? How has the public responded to the emergence of these technologies and how has the company responded to negative public sentiment? And what might this tell us about the future development and marketing trajectory of other robotic platforms with potential military applications? In order to get at some of these issues, we have conducted an examination of Twitter content that mentions Spot or its predecessors, dating back to 2007. An analysis of this data and the sentiments expressed in it toward Spot and its predecessors forms the next section of the article.

## Twitter analysis

### Data and method

Reactions to Boston Dynamics robots can be found across major social media platforms (e.g. Facebook) and online media platforms that allow social engagement with content (e.g. YouTube’s comment and reaction system). We chose to analyse data from Twitter because the platform allows researchers to capture data over a long time-period using their API. In total 117,215 tweets were collected spanning from May 2007 to August 2020 using software that queried Twitter’s API. We retrieved tweets that mentioned Boston Dynamics (or their Twitter account) with general terms related to robotic quadrupeds (i.e. ‘quadruped’, ‘quadrupedal’, ‘dog’, and ‘dogs’) or specific quadrupeds produced by Boston Dynamics (i.e. ‘spot’, ‘spotmini’, ‘alphadog’, ‘littledog’, ‘bigdog’, ‘ls-3’, ‘cheetah’).^1^ To avoid building a corpus mostly consisting of duplicated texts of viral tweets, retweets were excluded. 75.9 per cent of the tweets captured were classified as English-language tweets and these 88,970 tweets were the basis for our subsequent analysis.^2^

We investigated public reactions on Twitter systematically to answer three broad questions. Each question, in turn, required different analytic techniques. Firstly, we wanted to quantify mentions of Boston Dynamics robotic quadrupeds on Twitter over time. The number of tweets were aggregated for each month to identify periods with large numbers of reactions and these were analysed more closely to determine key drivers of public reaction. Secondly, we wanted to quantify ‘sentiment’ of tweets over time and assess perceptions of negativity associated with Boston Dynamics quadrupeds. Sentiment was quantified using Vader (Hutto and Gilbert 2014) to estimate the proportions of positive, neutral and negative sentiment over time. Thirdly, sentiment analysis is a somewhat blunt tool for understanding discourse, so we wanted to investigate the nature of ‘sentiment’ towards Boston Dynamics quadrupeds. To do this we examined prevalent words in the corpus that were influencing positive and negative sentiment scores using *concordancing*, a discourse analytic technique with a long history in textual scholarship closely associated with corpus linguistics (McCarthy and O'Keeffe, 2010). Concordancing allows systematic inspection of patterns of use of words (Baker 2006) and is an efficient way to represent the many occurrences of specific words or phrases in a corpus in their textual context and analyse patterns of usage and meaning. The concordance analysis was useful to understand features of discourse about Spot and its predecessors and several qualifications of the sentiment estimates.

### Analysing Twitter mentions over time

**Fig. 1:**
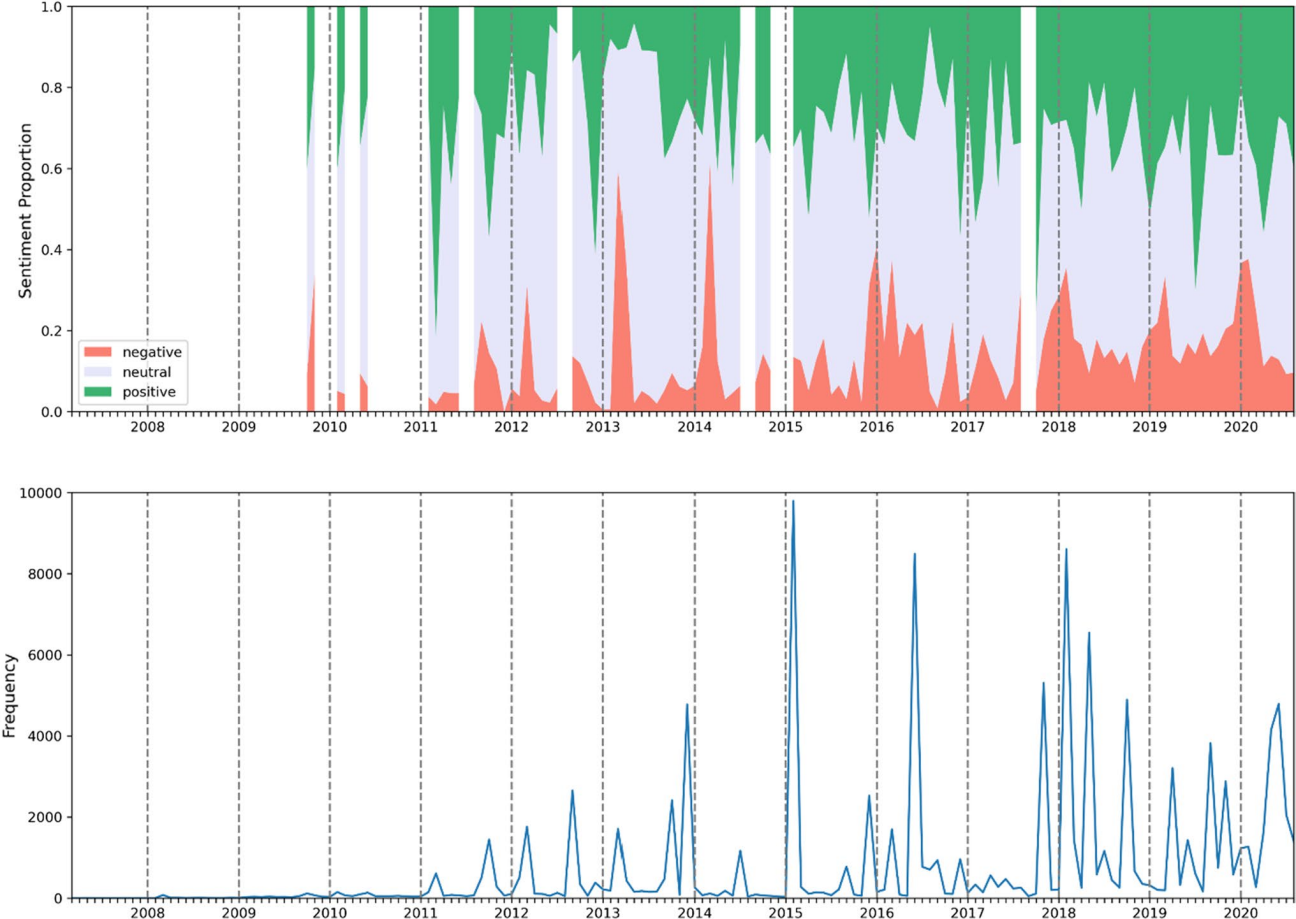
Twitter mentions and sentiment of Boston Dynamics quadrupeds 2007–2020. Sentiment proportions are only shown for months with at least 50 tweets.

The tweets were aggregated for each month and the most frequent months were examined more closely to understand the content of the tweets and key drivers of social media reactions (see Fig. 1 and Table 1). This analysis provided a data-driven method to identify key time periods of public attention on Boston Dynamics quadrupeds and this informed the construction of a timeline charting the development of robotic quadrupeds (Moses and Ford 2020). In most instances, months with the largest numbers of mentions coincided with video releases or media coverage. In an indication of the strong reaction that videos featuring Spot elicit on social media and the role of Boston Dynamics in driving this attention, eight out of the ten most frequent months coincided with videos released by Boston Dynamics (see Table 1). The remaining two most frequent months coincided with news stories about Spot, including the news that Alphabet (Google’s parent company) had purchased Boston Dynamics in December 2013 and media reports and videos in May 2020 about Spot being used in New Zealand to herd sheep and in Singapore to broadcast COVID-19 social distancing rules in a public park.

**Table 1:** Months with most tweets

Month	Tweets	Video or media reports during month
February, 2015	9797	‘Introducing Spot Classic (previously Spot)’ video
February, 2018	8605	‘Hey buddy can you give me a hand’ video
June, 2016	8491	‘Introducing Spot (previously SpotMini)’ video
May, 2018	6546	‘Spot Autonomous Navigation’ video
November, 2017	5310	‘The New Spot’ video
October, 2018	4893	‘UpTown Spot’ video, ‘Spot Robot Testing at Construction Sites’ video
June, 2020	4791	‘With you, Spot can’ video – list price for Spot
December, 2013	4774	Media reports about Alphabet purchasing Boston Dynamics
May, 2020	4152	Media reports about Spot being used in Singapore and New Zealand
September, 2019	3822	Announcing Spot for lease – ‘Spot Launch’ video

Media coverage indicated the success of Boston Dynamics’ marketing; video releases were amplified through mainstream media (e.g. CNN, The Guardian, New York Times) and technology-focused media (e.g. Wired, The Verge, Engadget, Gizmodo). Twitter users were often tweeting about media reporting of Boston Dynamics’ videos and frequently reproduced headlines and other text from these stories.

### Analysing sentiment over time

Sentiment of the captured English-language tweets was quantified using Vader, which derives its sentiment score based on a human-scored lexicon and ‘rules that embody grammatical and syntactical conventions for expressing and emphasizing sentiment intensity’ (e.g. negation) (Hutto and Gilbert 2014). Vader was developed to be used with social media texts and has been shown to perform well on a variety of text types and against other methods (Ribeiro et al*.* 2016; Hutto and Gilbert 2014). Vader’s open-source software allowed inspection of the algorithm and words underpinning the sentiment score.^3^

The overall sentiment scores that Vader derives for a text are interpreted as indicating positive, neutral or negative sentiment and these categories were used to assess the nature of the reaction to Boston Dynamics quadrupeds over time. Overall, in 88.0 per cent of months (103 of 117), more tweets expressed positive sentiment rather than negative sentiment. Comparison between frequency of mentions and sentiment for specific months indicated that the viral nature of the videos was often related to positive or negative sentiment during that month (see Fig. 1). For example, the month with the second highest number of mentions of Boston Dynamics quadrupeds, February 2018, coincided with the release of the video ‘Hey buddy can you give me a hand?’, which depicted two Spot devices opening and navigating a closed door. Tweets during the month this video was released tended to express negative sentiment (35.5 per cent) rather than positive sentiment (28.0 per cent). In contrast, in April 2020 when tweets were often reacting to media reports about the use of Spot to triage hospital patients and reduce the risk of medical workers contracting COVID-19, 55.8 per cent of tweets expressed positive sentiment. Our analysis of the nature of this sentiment adds insight to these blunt quantifications.

### Analysing the nature of sentiment

**Fig. 2:**
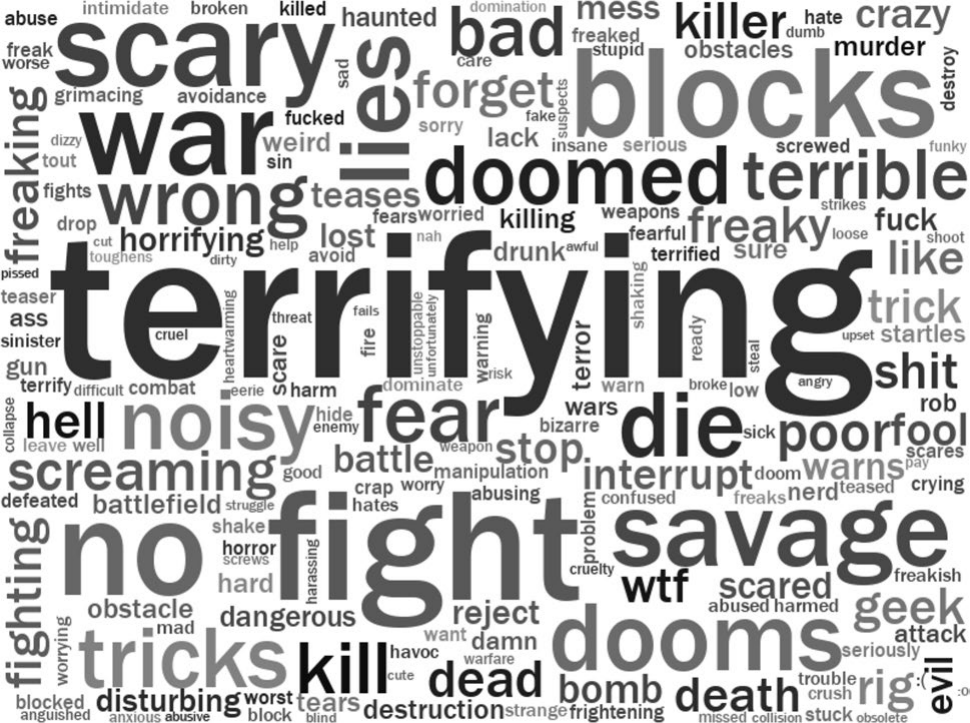
Words associated with negative reactions Boston Dynamics quadrupeds

**Fig. 3:**
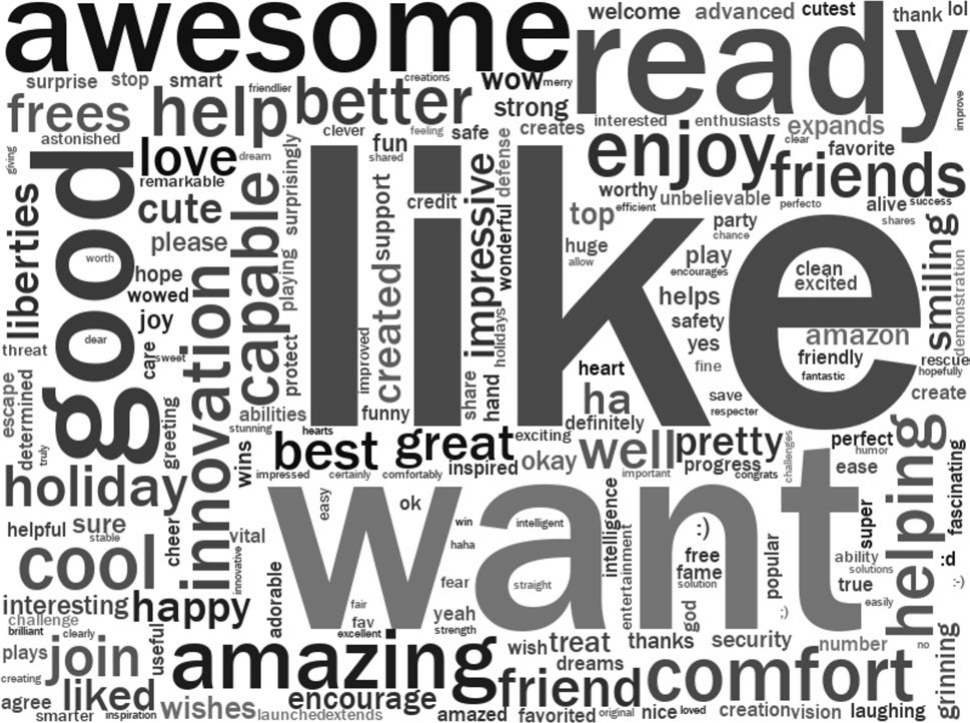
Words associated with positive reactions to Boston Dynamics quadrupeds

The most prevalent words in Vader’s lexicon influencing negative and positive sentiment scores are shown in Figs. 2 and 3. The size of each word in the word cloud indicates its prevalence in the corpus. The darkness of the text signifies its influence on the sentiment score (i.e. light gray = small influence on sentiment, dark grey or black = large influence). Further analysis of these words was undertaken to both understand the nature of the categories signified by ‘negative and ‘positive’ sentiment and to validate Vader’s sentiment scores. In the case of words associated with negative reactions to Boston Dynamics quadrupeds, we noticed that the most prevalent words could be loosely grouped into different semantic categories: fear (terrifying, scary, horrifying, fear), war, death and destruction (war, fight, fighting, battle, kill, die, dead, death, savage), dystopia (doomed, dooms), and negative moral evaluations (bad, wrong, evil). For words associated with positive reactions to Boston Dynamics quadrupeds there were a set of adjectives associated with positive evaluation (awesome, cool, good, great, amazing, impressive, better, best), helping (help, helping), technology (innovation), and signalling positive stance (like, love). However, it was important to validate that these semantic categories were meaningful and to investigate the use of words that did not clearly fit categories (e.g. blocks, lies, tricks, liberties, well, pretty, enjoy, friend, capable, comfort, ready).

**Fig. 4:**
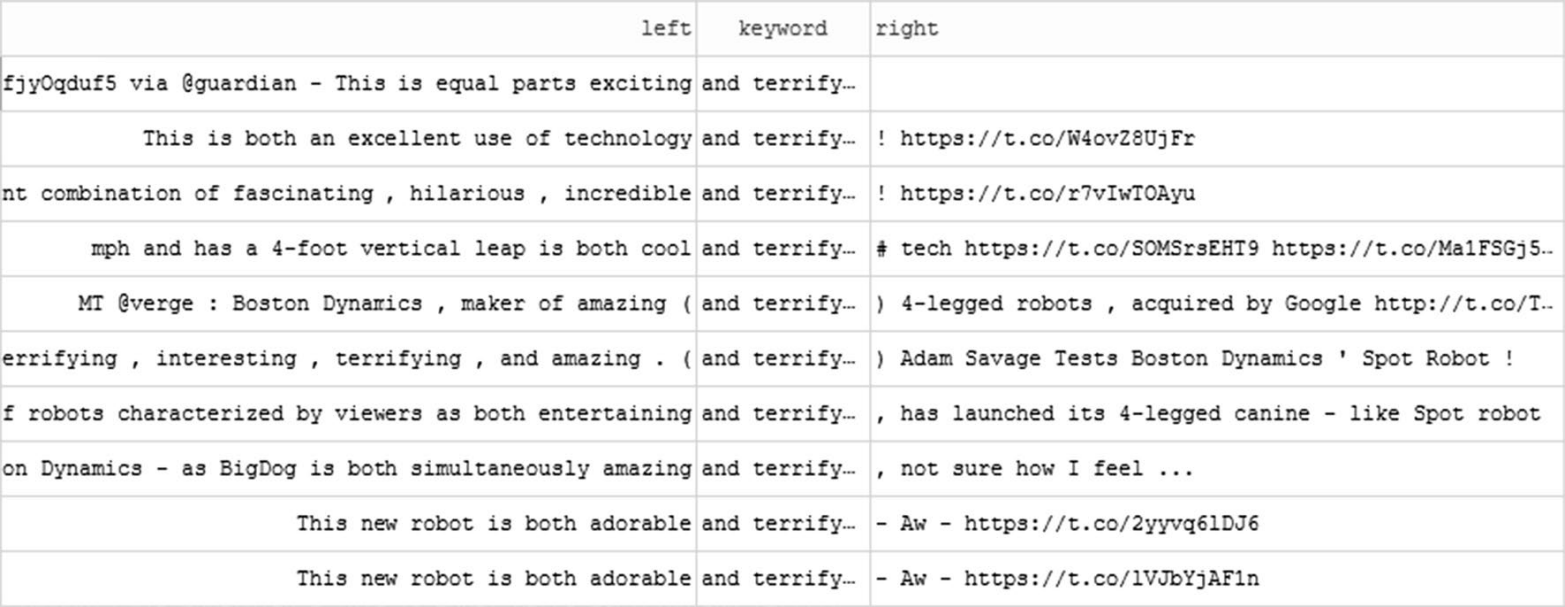
Excerpt of concordance showing 'and terrifying'

Our understanding of discourse about Boston Dynamics robotic quadrupeds was enriched through systematic analysis using concordancing. Our analysis also added to understanding of Vader’s sentiment measurement and it was found that sentiment scores exaggerated positive sentiment and underestimated negative sentiment due to recurring features of discourse. Firstly, it was common to mix words influencing positive and negative sentiment scores suggesting ambivalence about this fascinating but potentially concerning technology. For example, Fig. 4 shows an excerpt of the concordance for ‘and terrifying’ and the variety of positive words (‘exciting’, ‘excellent’, ‘fascinating’, ‘hilarious’, ‘incredible’, ‘cool’, ‘amazing’, ‘entertaining’, ‘adorable’) juxtaposed to ‘terrifying’. This expression of ambivalence about the robots was common (e.g. ‘Awesome and scary’, ‘adorable, yet horrifying’, ‘fear and astonishment’) and the compounding of sentiment scores across words in a tweet tends to underestimate the amount of negativity across the corpus.

**Fig. 5:**
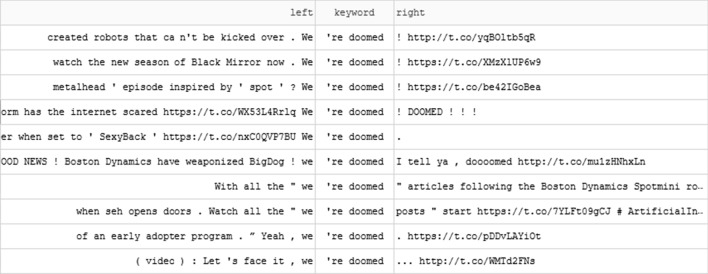
Excerpt of concordance showing recurring pattern 'we’re doomed'

The ambivalence demonstrated by contrasts between positive and negative sentiment-laden words relates to a second finding: there were certainly clear cases of positivity and negativity, but dark humour and sarcasm were recurring features of discourse about the robots. The negativity we quantified was often expressed using dark humour. The concordance in Fig. 5 shows examples of ‘we’re doomed’ and many of these examples are attempts at humour (e.g. ‘Yeah, we’re doomed’, ‘we’re doomed I tell ya, doooomed’), including the use of ‘we’re doomed’ in response to a video of Spot dancing to Justin Timberlake’s song ‘Sexyback’. When humour was expressed using sarcasm the accuracy of sentiment measures was limited. For example, the following quote was scored with positive sentiment based on the repeated use of ‘great’:

Great, just great. According to this “Planet Earth” parody, herds of Boston Dynamics’ robot dogs (they’re moving in herds….they DO move in herds!) are destined to roam the Earth once the era of human dominance comes to an end.

This was a common pattern for adjectives expressing positive sentiment like ‘great’ and ‘awesome’. Sarcasm is a well-known challenge for sentiment classification as textual features do not reliably indicate meaning or intent (Liu 2020). The recurring use of sarcasm we observed is likely to overestimate positive sentiment and underestimate negative sentiment.

**Fig. 6:**
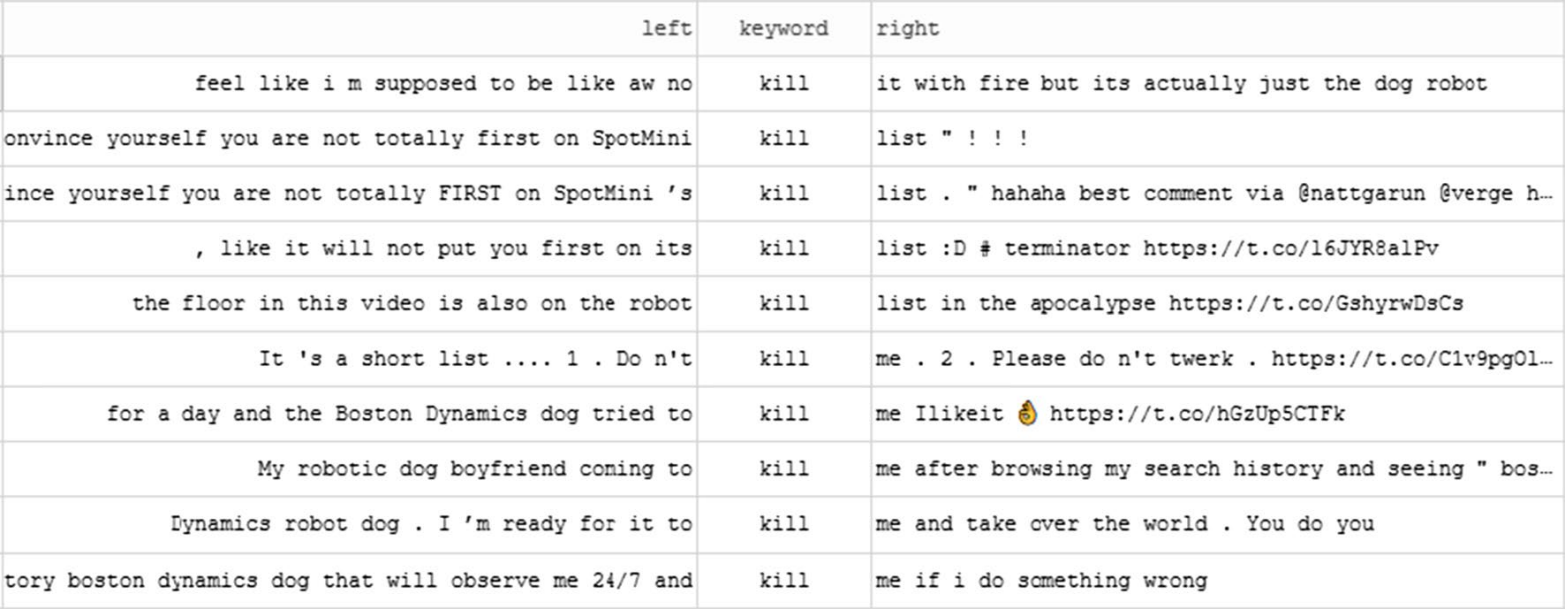
Excerpt of concordance of 'kill'

Thirdly, despite Spot and other Boston Dynamics quadrupeds not being used in combat or lethal roles, the tweets were rich with popular culture references about ‘killer robots’. While there were explicit references to words like ‘kill’ (see Fig. 6) and elaboration of the potential for Spot to be employed for lethal use (e.g. ‘Attach a gun and/or a bomb and you've got a weapon platform’), there were also references to popular culture expressions of the ‘killer robots’ trope. Negative sentiment (albeit often conveyed in a humorous way) is likely to underestimated because references to popular culture are not reflected in the sentiment tool’s lexicon. For example, although appealing to the ‘killer robot’ commonplace, the following tweets were scored with positive sentiment:

Unpopular opinion: I love the Boston Dynamics dog robots and I welcome them as our inevitable future overlords.

Great, @BostonDynamics made a robot dog that can open doors and @blackmirror is closer to being a documentary

Good question...why DO Google want Robots?? #Skynet

The ‘killer robot’ trope was a common reference in tweets, but as these examples indicate there were also specific mentions of ‘Black Mirror’ (1041 times) and the Black Mirror episode that featured a sprinting quadruped killer robot, namely ‘Metalhead’ (330 times). Tweets also referenced the movie Terminator (400 mentions) and entities and characters from the movie franchise, including ‘Skynet’, ‘Sarah Connor’ and ‘John Connor’. References to ‘Terminator’ and ‘SkyNet’ were one way in which people expressed suspicion of the connections between Boston Dynamics, the military, and then-owner Google.

Fourthly, elaboration of the representation of robot quadrupeds as dogs were common in tweets represented in the corpus. It was common for Twitter users to creatively employ words often associated with dogs (e.g. ‘fetch’, ‘fido’ ‘guard’, ‘woof’, ‘good boy’, ‘good robot’, ‘tricks’). References to ‘friend’ often alluded to the idea of dogs as ‘man’s best friend’ or ‘four-legged friend’, including creative variants like ‘military’s best friend’ and ‘Terminator’s new best friend’. Mentions of ‘like’ were often not straightforwardly expressing positive sentiment. Around one third of mentions of ‘like’ were in the forms ‘look/looks like’ or ‘walks like’, often referring to the dog-like appearance and gait of the robotic quadrupeds. Our analysis suggests Twitter users were both fascinated and disconcerted about the resemblance between animal and machine.

Finally, this analysis further demonstrated connections between social media texts and media reporting of Boston Dynamics. Media reports, reproduced or referenced on Twitter, were often sources of words influencing sentiment. Dark sarcastic humour and references to ‘killer robots’ appear to be common devices used by journalists to sensationalise and provoke interest and reactions in articles about Spot and other Boston Dynamics robotic quadrupeds. For example, of the 300 instances of ‘doomed’ in the corpus, 154 instances quoted the text ‘Let's face it, we're doomed’ in the course of sharing an Engadget article about Boston Dynamics’ Cheetah. The Engadget article began: ‘Let's face it, we're doomed as a species, because one day, Boston Dynamics is gonna unleash its army of DARPA-funded droids and wipe us all out’ (Cooper 2012).

This analysis revealed widespread interest in and fascination with Boston Dynamics quadrupeds. However, analysis also revealed ambivalence about these robots. Despite Boston Dynamics failure to secure contracts with the military and despite not being deployed as ‘killer robots’ there is ongoing suspicion about military associations and the potential for robot quadrupeds to be used as tools for violence and control. Key qualities of the discourse about Boston Dynamics robotic quadrupeds (dark humour, sarcasm, mixed sentiment, missed contextual references to popular culture) lead to fascinating patterns of misclassification of sentiment. Based on this close analysis, aggregated sentiment scores are likely to over-estimate positive and neutral sentiment. However, this is not to devalue the sentiment analysis: calculating positive and negative sentiment scores was a first step to approach closer analysis of the nature of sentiment. And, as the remaining sections show, sentiment is important to the funders, developers, and potential purchasers of these new robotic technologies.

## Robot quadrupeds as humanitarians

The founder and former CEO of Boston Dynamics, Marc Raibert, has made clear his desire to reduce the negative sentiment associated with Spot, telling a Wall Street Journal tech conference in 2019 that he was ‘really bothered’ by media coverage that referred to Boston Dynamics’ robots as ‘terrifying’. This negative sentiment, Raibert argued, stemmed from an excessive focus on dystopian portrayals of robots run amok in science fiction, and was insufficiently aware of the positive functions performed by the robots, which do not have ‘emotions and a personality and an ego — basically all the things that motivate malicious action in humans’ (DeKosta-Klipa 2019).

Raibert’s concerns about the negative sentiment associated with his company’s robots is clearly reflected in the shift in messaging and imagery being pushed by Boston Dynamics over the past few years. As we have shown above, much of the interest in Boston Dynamics’ robot quadrupeds has coalesced around the release of their videos via YouTube, peaking with the 2018 video, ‘Hey buddy can you give me a hand?’ (Boston Dynamics 2018a), which has over 140 million views at the time of writing. Given that reactions to this and earlier videos were often negative, there are indications Boston Dynamics have been consciously attempting to reshape attention in positive ways through the release of videos and other media interventions. Raibert himself acknowledged the importance of viral marketing and selective presentation of their robots in a 2013 interview, in which he argued that:

YouTube is a great way [to market Boston Dynamics]. You have complete control, so you show what you want to show, you don't show what you don't want to show, and we love that…I love YouTube; it's been a huge boon for us. I think it's had a big impact on our company... most people know who we are and what we do when we bump into them (Engadget 2013).

The sentiment analysis shows that this success was, at least in part, built upon the morbid fascination of the public with futures of robot warfare and dystopian sci-fi scenarios. Raibert’s understanding of how YouTube videos can be used to manipulate public sentiment toward the technology also suggests that there was (and is) a deliberateness to the image they have projected.

Boston Dynamics’ push toward commercial and industrial buyers has been accompanied by a clear desire to overcome these fear responses. In more recent marketing videos and activities from the company, we see the representation of Spot as benign and friendly technology designed to help and save human lives. In place of the previous images of BigDog hauling military gear or teams of Spots working together to open doors and move through buildings, we have seen increasing emphasis on the human-friendly domestic applications of Spot: fetching a drink for its owner in the home (Boston Dynamics 2016), performing a cute dance (Boston Dynamics 2018c), and working with Mythbusters host Adam Savage to pull a rickshaw (Adam Savage’s Tested 2020), for example. And on the industrial side, Boston Dynamics recommends that we ‘use Spot for documenting construction progress, reducing risk to people in dangerous environments, or other tasks that are too difficult for traditional automation’ (Boston Dynamics 2020b). Nuclear or chemical contaminated environments are particularly prevalent points of reference, as are collapsed buildings or other unstable environments in which search and rescue operations may need to be mounted.

But perhaps the most significant moment in the humanitarian framing of Spot arrived with the COVID-19 pandemic in early 2020. In 2020 Spot went viral, with Boston Dynamics demonstrating how it could be used in a healthcare role to ‘save lives, and fight COVID-19’ (Boston Dynamics 2020a). Stories appeared of Spot being used in Singapore to deliver a recorded message about sanitary practices in public parks (Tan 2020) and, more notably, of Spot carrying screens allowing doctors to interact with potential COVID patients without human contact at Brigham and Women’s Hospital in Boston (Statt 2020). At the Association of Unmanned Vehicles Systems International (AUVSI) Xponential conference in October 2020, Raibert again sought to emphasise the marketing upside of the humanitarian dimensions of Boston Dynamics’ robot quadrupeds, particularly in the wake of COVID-19:

The issue of whether people want to engage robots and whether they want to use them or not has had a different spin put on it since COVID’s been around because the opportunity to have a non-human go into a space and do a function and not have to expose the other people there or not have to expose the person who would have been the place of the robot I think has opened up a lot of people’s minds to how robots can play a role in their world (Raibert 2020).

It seems quite clear, therefore, that the current strategy of Boston Dynamics is to lessen the negative sentiment surrounding their technology in order to broaden its potential markets and normalize its presence in society. Emphasis on the ways in which these robots can be deployed to save human lives is central to that message and we are likely to see more of that in the coming years as the technology improves further.

The significance of this kind of humanitarian marketing of new technologies that have both civilian and military applications has been previously identified in the field of ‘humanitarian drones’ (Emery 2016; Jacobsen 2015). At the forefront of this area of research is the work of Kristin Bergtora Sandvik and a range of collaborators (Sandvik et al*.* 2014; Sandvik and Jumbert 2016; Sandvik and Lohne 2014), who have examined the emergence and implications of ‘humanitarian drones’ and ‘humanitarian technology’ more broadly. In a 2014 article by Sandvik and Lohne, the issue of technological crossover between humanitarian and military applications and the commercial imperatives underpinning that is addressed. Here, they argue that ‘a strong commercial logic underpins the push to reconceptualize drones as “humanitarian”’ (Sandvik and Lohne 2014, 149) and that ‘the UAV industry perceives the importance of presenting itself as humanitarian, as a means of gaining legitimacy’ (Sandvik and Lohne 2014, 150):

Thus, the concept of the humanitarian drone plays an important role – first, as vendors struggle to expand the market for UAVs by identifying new avenues of ‘humanitarian use’ for government customers, and, second, in relation to the general public, where vendors feel increasingly targeted by activists and critical news coverage (Sandvik and Lohne 2014, 150).

Following this line of thought, it is at least arguable that the movement toward a more humanitarian framing for Spot is geared not only toward appealing to the non-military commercial market, but also aids in the legitimization and normalization of the technology for military purposes. The COVID-19 pandemic, for example, has provided drone manufacturers the opportunity to legitimate the expanding civilian market for drones (Kaplan forthcoming). These insights on the significance of humanitarian framing of military technology appear on the face of it to fit very well with the story of Spot.

None of this is to say that new technology platforms, be they in the form of drones or robot dogs, have no actual or potential humanitarian use. The concern is that the foregrounding of the humanitarian uses constitutes an implicit and, at least in some cases, deliberate attempt to manage the negative sentiment attached to military or police uses of the same technologies. An examination of some of the underlying trends in military funding, development, and deployment of such technologies shows that there are real concerns about the ways in which public sentiment is manipulated in order to create an air of acceptability around these technologies and that this tends to occur with the melding of humanitarian and military aspirations to ‘save lives’.

## (Re-)Emerging military applications

Boston Dynamics received significant support from DARPA for the development of quadrupeds over the past 10–20 years, as well as the bipedal, humanoid robot now known as Atlas (Feldhaus Adams 2017). Despite the ‘do no harm’ clause in the lease contracts for Spot and the claims made that Boston Dynamics would not seek any further military contracts while they were under the ownership of Alphabet Inc (Markoff 2013), Marc Raibert has consistently left the door open to future work for and with the military. Following a TED Talk in 2017, Raibert responded to a question about military applications for Spot, saying that ‘the military has been a big funder of robotics. I don't think the military is the dark side myself, but I think, as with all advanced technology, it can be used for all kinds of things’ (TED 2017). At the 2019 Wall Street Journal tech conference he reaffirmed the position that Boston Dynamics ‘will “probably” have military customers’, but partnered this with the caveat that ‘the company would prohibit them, or any other customer, from using Spot to harm people’ (DeKosta-Klipa 2019). Again, the implication here is that this technology, whether in military or non-military settings, should be used to ‘save lives’ rather than take them.

The promise that military robots will ‘save lives’ should be understood as an example of the pervasive connections between humanitarianism and contemporary war. Whether this is framed in terms of ‘virtuous war’ (Der Derian and James 2001), ‘ethical war’ (Zehfuss 2018), or ‘humane war’ (Moyn 2021), the concern remains the same: that contemporary war is being justified and driven by humanitarian discourses that function to portray war as both increasingly safe for humans and geared toward moral imperatives. Principles associated with just war thinking, international humanitarian law, and humanitarian intervention have been integral to these developments over recent decades, generating ‘a form of war righteously pursued as superior precisely for being more humane, and one tolerated by audiences for that very reason’ (Moyn 2021, 6). There is, therefore, a need to start recognising and engaging with the ways in which ‘the politics of ethics’ (Zehfuss 2018) are playing out in the development of robotic and autonomous weapons technologies, as it is this framing (encapsulated in the promise that these robots will ‘save lives’) that is being used to both build acceptance of potentially autonomous military technologies and to resist calls for regulation or a ban under international law.

The intersection of military and humanitarian rationales is not new in thinking about the applications of robotic technologies. Going back to the late 1990s and early 2000s, some of the key thinkers from within the US military establishment and with ties to DARPA drew the same connections in their speculation about how robots might be used in military and non-military environments. Of particular interest here is the work of John Blitch, a former US Army Special Forces Commander who went on to be a program manager for DARPA’s Tactical Mobile Robotics Program from 1998–2001. During that period, Blitch published a series of articles detailing the potential uses of semi-autonomous robots for the future US military.

Emphasizing the challenges of urban warfare and terrorism, Blitch and his collaborators identified a range of issues that could be met by the deployment of robots, including challenging terrain, obstructed lines of sight, compromised communications, and presence of large numbers of non-combatants and media personnel in and around the battlefield (Krotkov and Blitch 1999). These environments would, according to Krotkov and Blitch, continue to present physical and psychological challenges to human war-fighters, and would present excessive risks that could be mitigated by the deployment of robotic systems. In these difficult environments, robots could ‘perform new classes of missions’, act as a ‘force multiplier’, and most importantly in the context of this paper, ‘save lives’ (Krotkov and Blitch 1999). Hence:

robotic systems can save lives, reduce casualties, and conserve personnel. Robots can do this directly by performing tasks that are hazardous to personnel and result in casualties. They can save lives less directly by providing soldiers and marines with information that allows them to perform their missions with less risk. Even less directly, a more effective force suffers fewer casualties, and to the extent that robots enhance force effectiveness, they will save lives (Krotkov and Blitch 1999, 771).

The dovetailing of military and humanitarian rhetoric around ‘saving lives’ is further reflected in Blitch’s later career, which has largely focused on the use of robots for urban search and rescue operations, which he terms ‘rescue robots’ (Blitch et al. 2000).

Blitch’s work in this area is fascinating in that it foregrounded the range of applications and challenges facing robot development that have persisted over the past twenty years and remain up to the present: The driving principle of taking humans out of the line of fire; the challenges of traversing diverse and dangerous terrains (including stairwells, a major point of emphasis for quadrupedal robotics companies); the teaming of robots with soldiers; their use as mobile sensors and surveillance units that can aid in the taking and holding of territory; the possibility of using autonomous robots in communications-denied environments; application of robots to scenarios of excessive risk such as mine clearance and bomb disposal; and the occasionally mentioned possibility of lethality or ‘neutralizing’ capabilities. All of these themes remain at the forefront of thinking about how quadrupedal robots can be used in the military, policing, and urban search and rescue, and in all cases the ‘saving lives’ theme is prevalent.

So what does all this mean for the current place of robot dogs in relation to military applications? In short, we are increasingly seeing evidence that DARPA’s investment in the technology is bearing fruit, although perhaps not yet for the company that led the charge. While Boston Dynamics remain open to future contracts with the military, another robotics company that specialises in quadrupedal robots is already showing a determination and capability to capture a significant place in that market themselves. Ghost Robotics emerged out of the General Robotics, Automation, Sensing & Perception Laboratory at Pennsylvania State University in 2016, going on the win the AUVSI Innovation Challenge for ‘Unmanned Systems — Defense. Protection. Security (USDPS)’ in 2018. Since then, Ghost’s ‘Vision 60’ quadruped has been trialled by the Australian Army (Keall 2019) and US Air Force (Hitchens 2020; Ackerman 2021), including a contract with the USAF in partnership with ARES Security that has a potential value of up to US$950 million (ARES Security 2020).

The CEO of Ghost Robotics, Jiren Parikh, has been far less coy about the potential military applications of his company’s quadrupeds than Boston Dynamics, commenting in early 2020 that the Vision 60 quadrupeds ‘can be used for anything from perimeter security to detection of chemical and biological weapons to actually destroying a target’ (Hitchens 2020). At the October 2021 Association of the United States Army (AUSA) conference in Washington DC, Ghost Robotics exhibited the Vision 60 fitted with a remote-controlled sniper rifle ‘payload’ produced by gun manufacturer SWORD International, triggering a strong negative response on Twitter (Ghost Robotics 2021a, 2021b). In a follow-up interview, Parikh played down the autonomy of the Vision 60, claimed to support the regulation of LAWS, and played up the potentially life-saving qualities of his company’s robots, remarking that ‘if we've saved one life because these people had our robot when they needed it, I think that's something to be proud of.’ Parikh also put public negativity towards robotic quadrupeds down to the influence of popular culture and claimed that ‘we will get used to these robots just like armed drones, they just have to be socialized’ (Ackerman 2021).

The case of Ghost Robotics illustrates how, as versatile ‘platforms’, robotic quadrupeds can be easily adapted using both hardware and software capabilities to a range of military and non-military tasks. They could ‘save lives’ by performing tasks in dangerous and compromised environments, but could also be set up to function as scouting, surveillance, and sniping teams for military units, or even as lethal autonomous weapons systems. The interplay and blurring of lines between these different dimensions raises serious questions about how public sentiment is being managed and manipulated as debates over the regulation of such technologies continues in a range of forums.

## Conclusion

Lifting the flap on Boston Dynamics’ Spot turns out to be quite a revealing exercise. Questions around funding, development, marketing, public perception, and legitimation of these quadrupedal robots all appear in intersecting ways, telling us quite a lot about the emergence of new military technologies and the management of the commercial and public aspects of that. Our sentiment analysis of Twitter responses to robotic quadrupeds provides an insight into the mix of terror and awe that these robotic technologies inspire in the public mind. While distinguishing the sentiment is often challenging, it is clear that videos of Spot have generated intense public interest and recurring negative reactions.

Both Boston Dynamics and their government and corporate funders seem to have a keen awareness of the importance of public sentiment toward their robot dogs and significant efforts are made to emphasize the ways in which they could ‘save lives’ in order to counteract the persistent sense of terror that they provoke in the public imagination. The promise that these robots could make war more humane and could contribute to a wide range of humanitarian and military missions is clearly central to the ongoing attempt to ‘socialize’ them. As Sandvik and Lohne (2014) point out, the two key outcomes of this process are the expansion of markets for the producers of the new technologies and the legitimation of those technologies through focus on life-saving commercial and humanitarian applications. If Spot is here to save lives, both in the battlefield and in civilian scenarios, then the likelihood of people being terrified of it or generating pressure to have the technology regulated or banned is reduced. These trends will require close and careful attention as international debates over the future of such technology continue.

## Footnotes

1 The search query used was: ("boston dynamics" OR @bostondynamics) (dog OR dogs OR spot OR spotmini OR alphadog OR littledog OR bigdog OR ls3 OR ls-3 OR quadruped OR quadrupedal OR cheetah).

2 Language detection was performed using the langdetect library (Danilák, 2020). After English-language texts, Japanese, Spanish, French and Russian the next more common languages represented in the corpus.

3 We modified Vader’s output so that words influencing the sentiment scores for each tweet in the corpus could be visualized. Doing so clarified that numerous occurrences of the word ‘dynamics’ appearing when *Boston Dynamics* was mentioned, were biasing sentiment scores in a positive direction. The words ‘Boston Dynamics’ (and the hashtags i.e. ‘#boston #dynamics’) were therefore removed from the tweets as a pre-processing step before calculation of sentiment scores.

## References

[CR1] Ackerman, Evan. 2021. Q&A: Ghost Robotics CEO on Armed Robots for the U.S. Military. Last modified October 18, 2021. https://spectrum.ieee.org/ghost-robotics-armed-military-robots

[CR2] Adam Savage’s Tested. 2020. Adam Savage's Spot Robot Rickshaw Carriage!. Last modified February 14, 2020. https://www.youtube.com/watch?v=zyaocKS3sfg&feature=emb_logo.

[CR3] Aker BP ASA. 2020. Meet Spot, the quadruped robot. Last modified February 11, 2020. https://www.youtube.com/watch?v=_uxm4iR39cc&feature=emb_title.

[CR4] ARES Security. 2020. ARES security awarded joint all domain command and control id/iq contract. Last modified August 4, 2020. https://aressecuritycorp.com/news/jadc2.

[CR5] Baker Paul (2006). Using corpora in discourse analysis.

[CR6] Blitch, John, Nahid Sidki and Tim Durkin. 2000. “Tactical mobile robots for urban search and rescue.” In *SPIE Proceedings Vol. 4024: Unmanned Ground Vehicle Technology II*, edited by Grant Gerhart, Robert Gunderson and Chuck Shoemaker. https://spie.org/Publications/Proceedings/Paper/10.1117/12.391630?SSO=1.

[CR8] Boston Dynamics. 2009. LittleDog. Last modified February 17, 2009. https://www.youtube.com/watch?v=UIipbi0cAVE.

[CR9] Boston Dynamics. 2012. Cheetah Robot runs 28.3 mph; a bit faster than Usain Bolt. Last modified September 6, 2012. https://www.youtube.com/watch?v=chPanW0QWhA.

[CR10] Boston Dynamics. 2013. Introducing WildCat. Last modified October 4, 2013. https://www.youtube.com/watch?v=wE3fmFTtP9g.

[CR11] Boston Dynamics. 2016. Introducing Spot (previously SpotMini). Last modified June 24, 2016. https://www.youtube.com/watch?v=tf7IEVTDjng.

[CR12] Boston Dynamics. 2018a. Hey Buddy, Can You Give Me a Hand?. Last modified February 13, 2018. https://www.youtube.com/watch?v=fUyU3lKzoio.

[CR13] Boston Dynamics. 2018b. Spot Robot Testing at Construction Sites. Last modified October 12, 2018. https://www.youtube.com/watch?v=wND9goxDVrY.

[CR14] Boston Dynamics. 2018c. UpTown Spot. Last modified October 16, 2018. https://www.youtube.com/watch?v=kHBcVlqpvZ8.

[CR15] Boston Dynamics. 2020a. *COVID-19 Response: Using Mobile Robots To Protect Healthcare Workers*. Last modified April 23, 2020. https://www.bostondynamics.com/COVID-19.

[CR16] Boston Dynamics. 2020b. With you, Spot can. Last modified June 17, 2020. https://www.youtube.com/watch?v=VRm7oRCTkjE.

[CR17] Boston Dynamics. 2021. Hyundai Motor Group completes acquisition of Boston Dynamics from Softbank. Last modified June 21, 2021. https://www.bostondynamics.com/hyundai-motor-group-completes-acquisition

[CR18] Brown, Mark. 2010. BigDog designers breed new four-legged robot. Last modified February 3, 2010. https://web.archive.org/web/20100309064803/https://www.wired.co.uk/news/archive/2010-02/03/bigdog-creators-awarded-contract-by-darpa.aspx.

[CR19] Carvin Stephanie, Williams Michael (2014). Law, science, liberalism and the American way of warfare: The quest for humanity in conflict.

[CR20] Cooper, Daniel. 2012. Boston Dynamics' Cheetah robot will hunt you down faster than any person (video). Last modified September 6, 2012. https://www.engadget.com/2012-09-06-boston-dynamics-cheetah-gets-faster.html.

[CR21] Danilák, Michal. 2020. *langdetect* (1.0.8). https://github.com/Mimino666/langdetect.

[CR22] DARPAtv. 2012. DARPA Legged Squad Support System (LS3). Last modified February 7, 2012. https://www.youtube.com/watch?v=xY42w1w0TWk.

[CR23] DeKosta-Klipa, Nik. 2019. The CEO of Boston Dynamics says it ‘really bothers’ him when people call their robots terrifying. Here’s why. Last modified October 28, 2019. https://www.boston.com/news/technology/2019/10/28/boston-dynamics-robots-terrifying.

[CR24] Der Derian James (2001). Virtuous war: Mapping the military-industrial-media-entertainment network.

[CR25] Dillon Michael, Reid Julian (2009). The liberal way of war: killing to make life live.

[CR26] Emery John (2016). The possibilities and pitfalls of humanitarian drones. Ethics & International Affairs.

[CR27] Engadget. 2013. Boston Dynamic's Marc Raibert backstage at Engadget Expand 2013. Last modified March 21, 2013. https://www.youtube.com/watch?v=dJVs9sUdFrQ.

[CR28] Feldhaus Adams, Rebecca. 2017. Backflipping Robot is a Giant Leap for Robot Kind. Last Modified November 17, 2017. https://www.npr.org/sections/thetwo-way/2017/11/17/564850067/back-flipping-robot-a-giant-leap-for-robot-kind.

[CR30] Ghost Robotics. 2021. Verizon CEO Hans Vestberg 5G Keynote CES '21: Ghost Q-UGV Mention from 5G Smart Cities Segment. Last modified January 12, 2021. https://www.youtube.com/watch?v=3icRite6RMk.

[CR31] Ghost Robotics. 2021. Latest lethality 6.5 #creedmoor sniper payload from @SWORDINT. Last modified October 12, 2021. https://twitter.com/Ghost_Robotics/status/1447699250570203137?s=20

[CR33] Hern Alex. 2015. US marines reject BigDog robotic packhorse because it's too noisy. Last modified December 30, 2015. https://www.theguardian.com/technology/2015/dec/30/us-marines-reject-bigdog-robot-boston-dynamics-ls3-too-noisy.

[CR34] Hern, Alex. 2017. Alphabet sells off 'BigDog' robot maker Boston Dynamics to Softbank. Last modified June 9, 2017.https://www.theguardian.com/technology/2017/jun/09/alphabet-sells-off-bigdog-robot-maker-boston-dynamics-to-softbank-google.

[CR35] Hibberd, James. 2017. 'Black Mirror' creator explains that 'Metalhead' robot nightmare. Last modified December 29, 2017. https://ew.com/tv/2017/12/29/black-mirror-metalhead-interview.

[CR36] Hitchens, Theresa. 2020. Innovators Are Air Force Target At ABMS Industry Day. Last modified January 27, 2020. https://breakingdefense.com/2020/01/air-force-seeks-innovators-at-first-abms-industry-day.

[CR37] Hutto Clayton J, and Eric Gilbert. 2014. Vader: A parsimonious rule-based model for sentiment analysis of social media text. n.d. http://comp.social.gatech.edu/papers/icwsm14.vader.hutto.pdf.

[CR38] Jabri Vivienne (2007). War and the transformation of global politics.

[CR39] Jacobsen Katja (2015). The politics of humanitarian technology: Good intentions, unintended consequences and insecurity.

[CR40] Jarmanning, Ally. 2019. Mass. State Police Tested Out Boston Dynamics’ Spot The Robot Dog. Civil Liberties Advocates Want To Know More. Last modified November 25, 2019. https://www.wbur.org/news/2019/11/25/boston-dynamics-robot-dog-massachusetts-state-police.

[CR41] Kaplan, Caren. Forthcoming. Everyday Militarisms: Drones and the Blurring of the Civilian-Military Divide During COVID-19. In Richardson, Michael and Pong, Beryl. eds. *Drone Aesthetics: War, Culture, Ecology*. London: Open Humanities Press.

[CR42] Keall, Chris. 2019. Australian Defence Force looks to integrate robots. Last modified November 27, 2019. https://www.nzherald.co.nz/business/australian-defence-force-looks-to-integrate-robots/NNRBU74FIGD3FKZTLOPGR7MULU.

[CR43] Krotkov Eric, Blitch John (1999). The defense advanced research projects agency (DARPA) tactical mobile robotics program. The International Journal of Robotics Research.

[CR44] Liu Bing (2020). Sentiment analysis: Mining opinions, sentiment, and emotions.

[CR45] Markoff, John. 2013. Google Adds to Its Menagerie of Robots. *N.Y. Times*. Last modified December 14, 2013. https://www.nytimes.com/2013/12/14/technology/google-adds-to-its-menagerie-of-robots.html.

[CR46] McCarthy Michael, O'Keeffe Anne, O'Keeffe A, McCarthy M (2010). Historical perspective: what are corpora and how have they evolved?. The Routledge Handbook of Corpus Linguistics.

[CR47] Moses Jeremy (2010). Liberal internationalist discourse and the use of force: Blair, Bush and beyond. International Politics.

[CR48] Moses Jeremy (2020). Why humanitarianism needs a pacifist ethos. Global Society.

[CR49] Moses, Jeremy and Geoffrey Ford. 2020. *Rise of the robot quadrupeds*. Last modified February 17, 2021. https://mappinglaws.net/rise-robot-quadrupeds.html.

[CR50] Moyn Samuel (2021). Humane: How the United States abandoned peace and reinvented war.

[CR51] Olinerd. 2008. Boston Dynamics Big Dog (new video March 2008). Last modified March 18, 2008. https://www.youtube.com/watch?v=W1czBcnX1Ww.

[CR53] Raibert Marc. 2020. Keynote address. *Association for Unmanned Vehicle Systems International (AUVSI) Xponential Conference 2020.*

[CR54] Ribeiro Filipe, Araújo Matheus, Gonçalves Pollyanna, Gonçalves Marcos André, Benevenuto Fabrício (2016). SentiBench - a benchmark comparison of state-of-the-practice sentiment analysis methods. EPJ Data Science.

[CR55] Rocos - Robot Operations Platform. 2020. Autonomous farm work - enter the robots. Last modified May 20, 2020. https://www.youtube.com/watch?v=RBLnAhzPpTQ.

[CR56] Sandvik Kirsten Bergtora, Jumbert Maria Gabrielsen, Karlsrud John, Kaufman Mareile (2014). Humanitarian technology: A critical research agenda. International Review of the Red Cross.

[CR57] Sandvik Kirsten Bergtora, Jumbert Maria Gabrielsen (2016). The good drone.

[CR58] Sandvik Kirsten Bergtora, Lohne Kjersti (2014). The rise of the humanitarian drone: Giving content to an emerging concept. Millennium - Journal of International Studies.

[CR59] Statt, Nick. 2020. Boston Dynamics’ Spot robot is helping hospitals remotely treat coronavirus patients. Last modified April 23, 2020. https://www.theverge.com/2020/4/23/21231855/boston-dynamics-spot-robot-covid-19-coronavirus-telemedicine.

[CR60] Stephen, Bijan. 2021. The NYPD deploys a robot dog again. Last modified February 24, 2021. https://www.theverge.com/2021/2/24/22299140/nypd-boston-dynamics-spot-robot-dog.

[CR61] SciNews. 2015. Marines testing Spot, the four-legged robot. Last modified September 22, 2015. https://www.youtube.com/watch?v=gqn_TyFMPOA.

[CR62] Tan, Cheryl. 2020. Robot reminds visitors of safe distancing measures in Bishan-Ang Mo Kio Park Last modified May 9, 2020. https://www.straitstimes.com/singapore/robot-reminds-visitors-about-safe-distancing-measures-in-bishan-ang-mo-kio-park.

[CR63] TED. 2017. Meet Spot, the robot dog that can run, hop and open doors. Last modified April, 2017. https://www.ted.com/talks/marc_raibert_meet_spot_the_robot_dog_that_can_run_hop_and_open_doors?language=en.

[CR64] Vincent, James. 2021. The French army is testing Boston Dynamics’ robot dog Spot in combat scenarios. Last modified April 7, 2021. https://www.theverge.com/2021/4/7/22371590/boston-dynamics-spot-robot-military-exercises-french-army.

[CR65] Zehfuss Maja (2011). Targeting: Precision and the production of ethics. European Journal of International Relations.

[CR66] Zehfuss Maja (2012). Contemporary Western War and the Idea of Humanity. Environment and Planning D: Society and Space.

[CR67] Zehfuss Maja (2018). War and the politics of ethics.

